# Supplementary material for: Neural dynamics of visual working memory representation during sensory distraction
Source: eLife. 2025 Jun 24;13:RP99290. doi: 10.7554/eLife.99290 (PMC12187136; doi:10.7554/eLife.99290)
Supplement: Supplementary file 1. [file elife-99290-supp1.docx]

**Supplementary Table 1.** FDR-corrected *p*-values corresponding to Figure 2d.

| Time points | No-distractor | Noise distractor | Orientation distractor |
| --- | --- | --- | --- |
| 4.8 - 7.2s | 0 | 0.032 | 0.031 |
| 4.8 - 9.6s | 0.081 | 0 | 0.052 |
| 4.8 - 12s | 0.092 | 0 | 0.052 |
| 4.8 - 14.4s | 0.081 | 0 | 0 |
| 4.8 - 16.8s | 0.031 | 0 | 0 |
| 7.2 - 9.6s | 0.078 | 0.523 | 0.375 |
| 7.2 - 12s | 0.031 | 0.067 | 0.2004 |
| 7.2 - 14.4s | 0 | 0.031 | 0.081 |
| 7.2 - 16.8s | 0.031 | 0 | 0.092 |
| 9.6 - 12s | 0.289 | 0.0667 | 0 |
| 9.6 - 14.4s | 0.158 | 0.067 | 0.031 |
| 9.6 - 16.8s | 0.031 | 0 | 0.081 |
| 12 - 14.4 | 0.289 | 0 | 0.648 |
| 12 - 16.8s | 0.031 | 0 | 0.067 |
| 14.4s - 16.8 | 0 | 0 | 0 |

**Supplementary Table 2.** FDR-corrected *p*-values corresponding to Figure 4b.

| Test | V1 | V2 | V3 | V3AB | V4 | IPS | LO1 | LO2 |
| --- | --- | --- | --- | --- | --- | --- | --- | --- |
| Noise-between baseline | 0 | 0 | 0 | 0 | 0.167 | 0 | 0 | 0.085 |
| Noise-within baseline | 0 | 0.388 | 0.139 | 0.337 | 0.2001 | 0.027 | 0.079 | 0 |
| Orientation-between baseline | 0 | 0 | 0 | 0 | 0.0598 | 0 | 0.0258 | 0.0454 |
| Orientation-within baseline | 0 | 0.176 | 0.2002 | 0.084 | 0.0258 | 0 | 0 | 0 |
| Noise generalization | 0.092 | 0 | 0.092 | 0 | 0.454 | 0.246 | 0.107 | 0.378 |
| Orientation generalization | 0.246 | 0.092 | 0.0896 | 0.118 | 0.763 | 0.246 | 0.551 | 0.251 |

**Supplementary Table 3.** FDR-corrected *p*-values corresponding to Supplementary Figure 6.

| Test | V1 | V2 | V3 | V3AB | V4 | IPS | LO1 | LO2 |
| --- | --- | --- | --- | --- | --- | --- | --- | --- |
| Naturalistic-between baseline | 0 | 0 | 0 | 0 | 0 | 0 | 0 | 0 |
| Naturalistic-within baseline | 0 | 0 | 0 | 0 | 0 | 0 | 0 | 0 |
| Orientation-between baseline | 0 | 0 | 0 | 0 | 0 | 0.010 | 0 | 0 |
| Orientation-within baseline | 0.031 | 0.033 | 0 | 0.0010 | 0 | 0.142 | 0.018 | 0 |
| Naturalistic generalization | 0.368 | 0.109 | 0 | 0.109 | 0.072 | 0.109 | 0.109 | 0.037 |
| Orientation generalization | 0.321 | 0.109 | 0.109 | 0.273 | 0.948 | 0.220 | 0 | 0.274 |
